# A Wavelet Bicoherence-Based Quadratic Nonlinearity Feature for Translational Axis Condition Monitoring

**DOI:** 10.3390/s140202071

**Published:** 2014-01-27

**Authors:** Yong Li, Xiufeng Wang, Jing Lin, Shengyu Shi

**Affiliations:** 1 School of Mechanical Engineering, Xi'an Jiaotong University, Xi'an 710049 China; E-Mails: liyongmec@stu.xjtu.edu.cn (Y.L.); wangxiufeng@mail.xjtu.edu.cn (X.W.); shi.shengyu@stu.xjtu.edu.cn (S.S.); 2 State Key Laboratory for Manufacturing Systems Engineering, Xi'an Jiaotong University, Xi'an 710049, China

**Keywords:** condition monitoring, wavelet bicoherence, quadratic nonlinearity, translational axis system

## Abstract

The translational axis is one of the most important subsystems in modern machine tools, as its degradation may result in the loss of the product qualification and lower the control precision. Condition-based maintenance (CBM) has been considered as one of the advanced maintenance schemes to achieve effective, reliable and cost-effective operation of machine systems, however, current vibration-based maintenance schemes cannot be employed directly in the translational axis system, due to its complex structure and the inefficiency of commonly used condition monitoring features. In this paper, a wavelet bicoherence-based quadratic nonlinearity feature is proposed for translational axis condition monitoring by using the torque signature of the drive servomotor. Firstly, the quadratic nonlinearity of the servomotor torque signature is discussed, and then, a biphase randomization wavelet bicoherence is introduced for its quadratic nonlinear detection. On this basis, a quadratic nonlinearity feature is proposed for condition monitoring of the translational axis. The properties of the proposed quadratic nonlinearity feature are investigated by simulations. Subsequently, this feature is applied to the real-world servomotor torque data collected from the X-axis on a high precision vertical machining centre. All the results show that the performance of the proposed feature is much better than that of original condition monitoring features.

## 1. Introduction

The translational axis is one of the most important subsystems in modern machine tools, which has been widely used for transforming rotational motion into linear motion, due to its advantages of low sensitivity to inertia variations, good positional accuracy and high driving speeds [1]. Starting from a new condition, each part of the translational axis system is properly manufactured and installed into the system without any errors, and is operated at a particular level of performance. As their service life progresses, faults such as material fatigue, abrasion or adhesion in the components always leads to a performance degradation, which means the parts can no longer meet their original service requirements [2]. This performance degradation of a translational axis system may cause some problems. First of all, it may produce distance errors between the tool tip and the workpiece in the machining process and thus lead to the loss of the product qualification [3,4]. Secondly, any faults occuring in components will lower the control precision [1,5]. It is, therefore, very essential to investigate the technology for translational axis system condition monitoring. Condition-based maintenance (CBM) has been considered as one of the advanced maintenance schemes to achieve effective, reliable and cost-effective operation of machines and systems [6]. In a CBM system a machine's condition before it fails can be monitored and predicted, and optimal maintenance actions can be scheduled to improve reliability and reduce the possibility of breakdowns [7]. In the past few years, condition monitoring methods for machine tools have been widely studied as the key research topics in CBM [8–10]. Ottewill and Orkisz [11] used synchronously averaged electric motor signals to monitor gearboxes, showing that this method was extremely adept at locating gear tooth defects. Hwang and Lee [12] studied a condition monitoring method based on the change likelihood of a stochastic model, which was successfully applied to the monitoring of machine condition and weld condition. Sinha and Elbhbah [13] focused on the monitoring of rotating machines by enhancing the computational effort in signal processing to reduce the number of sensors per bearing pedestal. Subsequently, Hassan and Bayoumi *et al.* [7] proposed a Fourier bicoherence-based index for health condition monitoring of helicopter drive trains. Yu [14] proposed a Generative Topographic Mapping (GTM) and contribution analysis-based method to perform machine health degradation assessment and monitoring. Generally, most of the current methods are based on identifying some significant change in vibrations of the system. However, current vibration-based maintenance schemes cannot be employed in the translational axis system directly, due to its complex structure and the inefficiency of the commonly used condition monitoring features [6,15]. In this paper, a wavelet bicoherence-based quadratic nonlinearity feature is proposed for translational axis condition monitoring by using the torque signature of the drive servomotor. Firstly, the quadratic nonlinearity of the servomotor torque signature is discussed, and then, a biphase randomization wavelet bicoherence is introduced for its quadratic nonlinear detection. On this basis, a quadratic nonlinearity feature is proposed for condition monitoring of the translational axis. The key idea is that as the performance of the translational axis degrades, the torque signature will tend to be more nonlinear, which will result in the generation of new frequencies [16,17]. Those new frequencies are phase coupled to the original characteristic frequencies of the translational axis, and the wavelet bicoherence is a sensitive detector for identifying such phase coupling [18]. The remaining parts of this paper are organized as follows: in Section 2, a theoretical basis is set up to show the quadratic nonlinearity of motor torque signal, under the fault condition of translational axis system. After that, a biphase randomization wavelet bicoherence technique is introduced in Section 3, and a quadratic nonlinearity feature is proposed for condition monitoring. The performance of the proposed quadratic nonlinearity feature is investigated through a simulation in Section 4. In Section 5, this feature is applied to the translational axis condition monitoring of a vertical machining centre in an in-service environment. Finally, conclusions are given in Section 6.

## 2. Quadratic Nonlinear Model of Servomotor Torque

**Figure 1.**
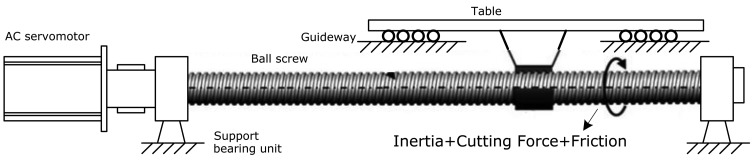
Illustration of the translational axis system.

As shown in Figure 1, the translational axis system is often composed of an AC servomotor, a ball screw, a table, a guideway and other rotating components (such as support bearing and reducer). These parts work cooperatively to guarantee the table is constrained to move axially on guideways once the servomotor torque overcomes the disturbance and table inertia. In order to calculate the torque of the AC servomotor, the three-phase AC currents of the servomotor are measured and converted into DC current by using the D-Q transformation as follows [19,20]:

(1) $$\left(\begin{array}{l} I_{q}\\ I_{d} \end{array}\right)=\sqrt{\frac{2}{3}}\left[\begin{array}{ccc} \cos n_{p}\theta & \cos(n_{p}\theta -\frac{2}{3}\pi ) & \cos(n_{p}\theta +\frac{2}{3}\pi )\\ \sin n_{p}\theta & \sin(n_{p}\theta -\frac{2}{3}\pi ) & \sin(n_{p}\theta +\frac{2}{3}\pi ) \end{array}\right]\;\left[\begin{array}{c} I_{u}\\ I_{v}\\ I_{w} \end{array}\right]$$

where *I_u_*,*I_v_* and *I_w_* are the *u*-, *v*- and *w*-phase AC current respectively, *n_p_* is the number of poles, *θ* is the relative angle between the *u* and Q-axes. The D-Q transformation method transforms rotating coordinates to the D-axis and Q-axis, in fixed coordinates, and the converted DC current *I_q_* is directly related to the motor torque, *I_d_* is maintained at zero.

During the cutting process, the measured disturbance torque is composed of frictional torque and the torque caused by the cutting force (as shown in Figure 1), which is proportional to the motor DC current *I_q_* according to the ratio of torque constant *δ_T_*. Hence, the total motor torque can be modeled as:

(2) $$T=\delta_{T}I_{q}=J\frac{d\omega }{dt}+T_{f}+T_{c}$$

where *I_q_* is the equivalent DC current of the drive motor, *δ_T_* is the motor torque constant, *J* is the equivalent total moment of inertia of the translational axis system, *ω* is the rotational speed of rotor, *T_f_* is the friction torque, and *T_c_* is the cutting torque.

When the table is fed steadily without cutting, the measured disturbance torque only represents the frictional torque of guideways, ball screw and other rotating components (such as support bearing and reducer). Then, *T_c_* = 0, and Equation (2) is modified as:

(3) $$T=\delta_{T}I_{q}=J\frac{d\omega }{dt}+T_{f}$$

During the assembly of machine tools, a preload must be exerted to minimize the clearance of the ball screw and support bearing to improve the dynamical operational behavior [21]. The increase of the preload will raise the stiffness of the ball screw and support bearing [22]. However, increasing the preload will also increase the friction on the ball screw and support bearing. During the serving cycle, various frictional components such as rolling, bore and sliding friction will lead to different faults such as material fatigue, abrasion or adhesion, and ultimately potentially result in additional torque. Hence, as shown in Equation (3), the torque under no load condition can be used as an attribute of the condition monitoring of the translational axis system [22]. Commonly, with the degradation of the translational axis system, the servomotor torque signature will tend to become more nonlinear. The nonlinearity mechanism is often very complex. In this article, the quadratic nonlinear (a strong indicator of nonlinear signal) of the torque signature is considered [23,24]. The quadratic nonlinear system response, *x*(*t*), for an input *y*(*t*) is:

(4) $$x(t)=ay^{2}(t)+by(t)+n(t)$$

where *a* and *b* are two constants that define the linear and nonlinear components of the system input and output relationship, *n*(*t*) is additive Gaussian white noise.

The degradation of the part in the translational axis system will lead to an additional friction. Suppose that the additional friction *y*(*t*) is a cosinusoid wave, and due to nonlinearity and harmonic production, a suitable input is two mixed cosinusoids:

(5) $$y(t)=T_{1}\cos(2\pi f_{1}t+\varphi_{1})+T_{2}\cos(2\pi f_{2}t+\varphi_{2})$$

To avoid the additional friction, an additional torque should be provided by the servomotor. Substituting Equation (5) into Equation (4) and applying trigonometric identities, the additional torque response is given as:

(6) $$\begin{array}{c} x(t)=bT_{1}\cos(2\pi f_{1}t+\varphi_{1})+bT_{2}\cos(2\pi f_{2}t+\varphi_{2})+a\frac{T_{1}^{2}}{2}\cos[2\pi (2f_{1})t+2\varphi_{1}]+a\frac{T_{2}^{2}}{2}\cos[2\pi (2f_{2}\\ +aT_{1}T_{2}\cos[2\pi (f_{2}-f_{1})+(\varphi_{2}-\varphi_{1})]+aT_{1}T_{2}\cos[2\pi (f_{2}+f_{1})+(\varphi_{2}+\varphi_{1})]+n(t) \end{array}$$

In the frequency domain, the continuous wavelet transform (CWT) spectrum of Equation (6) can be obtained as:

(7) $$\begin{array}{c} X[x(t)]=bT_{1}W_{\psi }(f_{1},t)e^{i\varphi_{1}}+bT_{2}W_{\psi }(f_{2},t)e^{i\varphi_{2}}+a\frac{T_{1}^{2}}{2}W_{\psi }(2f_{1},t)e^{i2\varphi_{1}}\\ +a\frac{T_{2}^{2}}{2}W_{\psi }(2f_{2},t)e^{i2\varphi_{2}}+aT_{1}T_{2}W_{\psi }(f_{2}-f_{1},t)e^{i(\varphi_{2}-\varphi_{1})}+aT_{1}T_{2}W_{\psi }(f_{2}+f_{1},t)e^{i(\varphi_{2}+\varphi_{1})}+N(f) \end{array}$$

where *W_ψ_*(*f*, *t*) is the CWT of cos(2*π ft*), *N*(*f*) is the CWT of the Gaussian noise *n*(*t*).

As shown in Equation (7), the nonlinearity signals at 2 *f*_1_ and 2 *f*_2_ are denoted as the harmonics, and those at *f*_2_ − *f*_1_ and *f*_2_+*f*_1_ are the nonlinear interaction components. In addition, the phase of the signals caused by the nonlinearity satisfies the relationship, *φ*_1_ + *φ*_2_ = *φ*_3_, where *φ*_1_ and *φ*_2_ are the phase of the two coupling components, *φ*_3_ is the phase of the new interaction component. This is the quadratic phase coupling (QPC) due to system nonlinear behavior, which provides a way to distinguish signals at a particular frequency. Because only the components contributed to the QPC are of interest, all other terms are neglected, and Equation (6) is modified as [18,24]:

(8) $$x(t)=bT_{1}\cos(2\pi f_{1}t+\varphi_{1})+bT_{2}\cos(2\pi f_{2}t+\varphi_{2})+aT_{1}T_{2}\cos[2\pi (f_{2}+f_{1})+(\varphi_{2}+\varphi_{1})]+n(t)$$

The simplified QPC model is employed widely for condition monitoring [18]. By checking the phase coupling frequency components, various faults can be recognized with high probability. Bispectrum and bicoherence are effective in detecting the QPC in this type of nonlinear signal [25,26], but, they are not appropriate for the signal associated with short-time duration nonlinear interactions. Recently, a wavelet bicoherence (WB) method combining benefits of both wavelet transform and bicoherence analysis is proposed for nonlinear and non-stationary signal analysis [27–29]. Compared with the Fourier bicoherence, WB has its advantage in preservation of temporal information, thus it is more suitable for the instantaneous servomotor torque analysis.

## 3. Estimation of Wavelet Bicoherence Based Quadratic Nonlinearity Feature

### 3.1. Biphase Randomization Wavelet Bicoherence

The WB is firstly introduced by van Milligen [30]. Given a signal *x*(*t*), the continuous wavelet transform (CWT) of *x*(*t*) is defined as the convolution of *x*(*t*) with the scaled and normalized wavelet. We write:

(9) $$W_{\psi }(f,t)=\frac{1}{\sqrt{\left|s\right|}}\int_{-\infty }^{\infty }x(t^{'})\psi^{*}(\frac{t^{'}-t}{s})dt^{'}$$

where the * indicates the complex conjugate, *ψ*(*t*) is an admissible mother wavelet, *s* is the scale variable, *t* is the time shift variable, *f* is the equivalent Fourier frequency.

In an analogous definition to the usual Fourier bispectrum, the wavelet bispectrum is defined as follows [30]:

(10) $$B_{W,T}(f_{1},f_{2})=\int_{T}W_{\psi }(f_{1},t)W_{\psi }(f_{2},t){W_{\psi }}^{*}(f_{3},t)dt$$

where the * indicates the complex conjugate, *T* is the finite time interval of the signal, frequency values *f_1_*, *f_2_* and *f_3_* satisfy the relationship *f_3_* = *f_1_* + *f_2_*. The wavelet bispectrum is a complex value. So, it can be also expressed by its magnitude *A*(*f_1_*, *f_2_*) and biphase *φ*(*f_1_*, *f_2_*) as follows:

(11) $$B_{W,T}(f_{1},f_{2})=A(f_{1},f_{2})e^{i\varphi (f_{1},f_{2})}$$

where the biphase *φ*(*f_1_*, *f_2_*) can be calculated by, *φ*(*f*_1_, *f*_2_) = *φ*_1_(*f*_1_) + *φ*_2_(*f*_2_) − *φ*_3_(*f*_3_). Here, *φ*_1_(*f*_1_), *φ*_2_(*f*_2_) and *φ*_3_(*f*_3_) are phases uniformly distributed within (−*π*, *π*], obtained by CWT.

Conventional wavelet spectrum based WB is incapable of eliminating the spurious peaks coming from long coherence time waves and non-QPC waves [23,31]. As a result, direct application may cause incorrect results. To overcome this problem, a biphase randomization wavelet bispectrum is introduced as [32,33]:

(12) $${B^{\prime }}_{W,T}(f_{1},f_{2})=E\left(\int_{T}W_{\psi }(f_{1},t)W_{\psi }(f_{2},t){W_{\psi }}^{*}(f_{3},t)e^{iR\varphi (f_{1},f_{2})}dt\right)$$

where *e^iRφ^*^(^*^f^*^1,^*^f^*^2)^ is the biphase randomization term, *φ*(*f_1_*, *f_2_*) is the biphase obtained by Equation (11), *R* is a random variable distributed within (−*π*, *π*], *E*[ ] denotes an average operator.

After normalization, the biphase randomization wavelet bicoherence (BRWB) is given as [32]:

(13) $$b_{W,T}^{'2}(f_{1},f_{2})=E\left(\frac{{\left|B_{W,T}^{'}(f_{1},f_{2})\right|}^{2}}{{\int_{T}\left|W_{\psi }(f_{1},t)W_{\psi }(f_{2},t)\right|}^{2}dt\int_{T}{\left|W_{\psi }(f_{3},t)\right|}^{2}dt}\right)$$

where *E*[] denotes an average operator. BRWB characterizes the QPC among different frequency components of the signal. The peaks in the BRWB indicate the phase and frequency coupling at bifrequency (*f_1_*, *f_2_*) during the time interval *T*, and the values of the peaks are bounded between 0 and 1. By using the proposed BRWB, the spurious bicoherence coming from long coherence time waves and non-QPC waves can be eliminated automatically.

The summed feature of BRWB (SBRWB feature) is used commonly to measure the distribution of phase coupling, which is defined as [30]:

(14) $$S_{\text{BRWB}}=\int_{f_{2}}b_{W,T}^{'}(f_{1},f_{2})df_{2}$$

At the same time, a BRWB-based quadratic nonlinearity feature, namely the maximum eigenvalue of the BRWB (MEBRWB feature) [29,34], is proposed to make condition monitoring of the translational axis system more convenient. Since the BRWB is a symmetrical matrix to the main diagonal, denoted as $\left[M]=b_{W,T}^{'}(f_{1},f_{2}\right)$, it can be decomposed as:

(15) $$\left[M][V_{k}]=\lambda_{k}[V_{k}\right]$$

where *λ_k_* is the eigenvalue, [*V_k_*] is the eigenvetor corresponding to *λ_k_*. The value of eigenvalue is proportional to the amount of correlation in the direction of their associated eigenvectors, and the maximum eigenvalue linearly increases with the increase of the correlation strength in a cluster. Therefore, the single-value MEBRWB feature can be defined as a global phase coupling feature of the BRWB.

### 3.2. Estimation of BRWB and Its Quadratic Nonlinearity Features

**Figure 2.**
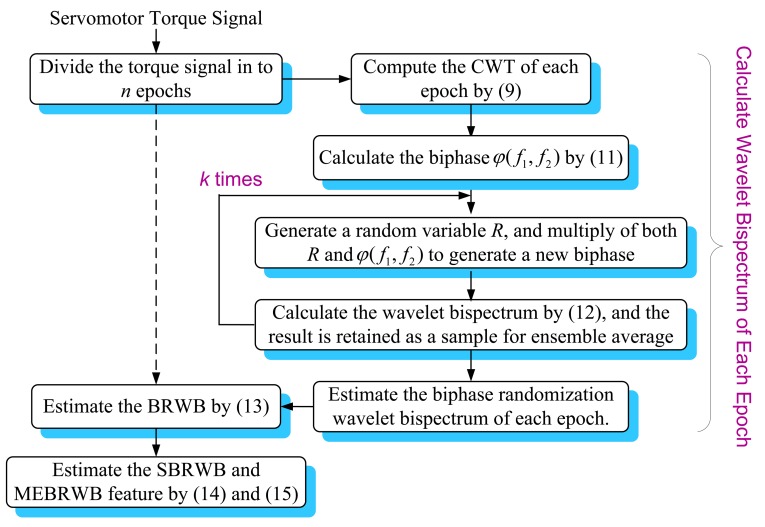
Flow chart of the proposed BRWB and its feature estimation.

The estimation of the BRWB, the SBRWB and MEBRWB features is detailed in the following steps as illustrated in Figure 2.

Step 1: Divide the servomotor torque signal into *n* epochs.
  Generally, in computing of BRWB, the signal with long data record length is firstly divided into a series of epochs, and then, the reliable bicoherence estimation of the signal is obtained by averaging the BRWB of all epochs. In this study, the BRWB of torque signal is calculated based on smallest meaningful signal segment, due to its non-stationary property. Because that the signal segment of 1/*F_PFL_* (*F_PFL_* is the passing frequency of ball screw lead) length often covers all characteristic frequencies of translational axis system, the torque signal is divided into *n* epochs with each period equal to1/*F_PFL_*, with the overlap of 75% [29].
Step 2: Compute the wavelet transform *W_ψ_*(*f*, *t*) of each epoch by Equation (9).
  For the torque signals, impulse components often correspond to the degradation of the translational axis system, thus the basic wavelet used for feature extraction should be similar to an impulse [35]. For this reason, the Morlet wavelet is chosen for feature extraction in this study. Since the wavelet bicoherence calculation involves three scales, the choice of wavelet parameter should tradeoff between the time width of analysis wavelet function and best suited individual scale. Here, takes *ω*_0_ to 6 to satisfy the admissibility condition, and takes *σ* within 3 to 8 to obtain the appropriate half power time-width of the mother wavelet.
Step 3: Calculate the biphase *φ*(*f*_1_, *f*_2_) of each epoch by using Equation (11).
Step 4: Generate a random variable *R* within(−*π*, *π*], and multiply of both *φ*(*f*_1_, *f*_2_) and *R* to generate a new biphase *R φ*(*f*_1_, *f*_2_).
  In practice, signals often have very long coherence time, which makes the biphase component dependent over each epoch. The biphase component dependent over each epoch may cause spurious bicoherence. In this step, a biphase randomization term *e^iRφ^*^(^*^f^*^1,^*^f^*^2)^ is generated to damage the phase component dependent condition of each epoch by using the new biphase *R φ*(*f*_1_, *f*_2_). At the same time, the biphase randomization term is also used to eliminate the spurious bicoherence coming from non-QPC waves.
Step 5: Calculate the wavelet bispectrum by Equation (12).
  In this step, the biphase randomization term *e^iRφ^*^(^*^f^*^1,^*^f^*^2)^ generated by Step 4 is introduced for the wavelet bispectrum calculation, and the result is retained as the sample for the reliable bispectrum estimation. Actually, *k* data samples can be obtained after *k* times of cycles of the Step 4 and Step 5. *k* is taken 100 to keep the balance between a satisfied calculation result and an acceptable calculation time. Thus, 100 data samples can be obtained for reliable bispectrum estimation of this signal epoch, and each data sample is calculated by different biphase randomization term *e^iRφ^*^(^*^f^*^1,^*^f^*^2)^.
Step 6: Estimate the biphase randomization wavelet bispectrum of each signal epoch.
  In this step, the biphase randomization wavelet bispectrum of this signal epoch is estimated by ensemble average operation of the 100 data samples obtained in Step 5. The spurious bispectrum coming from non-QPC waves can be eliminated by the ensemble averaging operation. After normalization, the BRWB of this signal epoch can be obtained, and the result is retained as the samples for reliable BRWB estimation. From this step, *n* BRWB data samples can be obtained for reliable BRWB estimation.
Step 7: Estimate the BRWB by Equation (13).
  Since *n* signal epochs are used for BRWB estimation, here, the reliable BRWB is estimated by averaging *n* BRWB data samples obtained in Step 6. The biphase component of each BRWB sample has been made independent over each other in previous steps. Thus, by the average operation, the spurious bicoherence coming from long coherence time waves can be eliminated automatically. Commonly, 10–20 signal epochs are sufficient to obtain a reliable BRWB estimation [32].
Step 8: parameter estimation by Equations (14) and (15).
  The SBRWB feature is calculated by Equation (14), and an eigenvalue series can be obtained from the Equation (15). By extracting the maximum value of this series, a single-value MEBRWB feature is obtained for global QPC measurement of the torque signal.

## 4. Simulations

**Figure 3.**
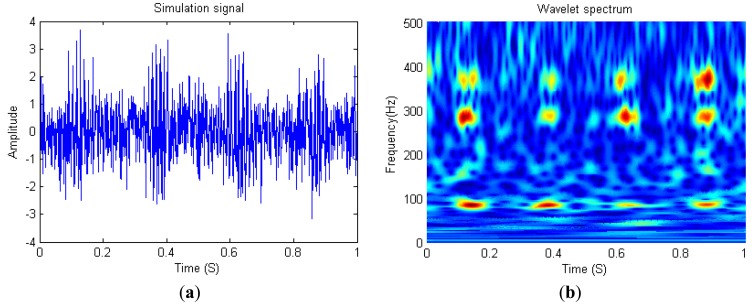
Simulation signals (**a**) and its wavelet scalogram (**b**).

According to the QPC model introduced in Equation (8), the simulation signal model is as follows [24,35]:

(16) $$\begin{array}{c} x(t)=[\cos(2\pi f_{1}t+\varphi_{1})+\cos(2\pi f_{2}t+\varphi_{2})\\ +A(\cos(2\pi (f_{1}+f_{2})t+(\varphi_{1}+\varphi_{2})))+(1-A)(\cos(2\pi (f_{1}+f_{2})t+\varphi_{3}))]e^{\left[-{\left(t-(nT+0.125)\right)}^{2}/(1/600)\right]}+n(t) \end{array}$$

where *f_1_* and *f_2_* are the coupled frequency, *φ*_1_ and *φ*_2_ are the initial phase distributed within (−*π*, *π*] uniformly, *n*(*t*) is white Gaussian noise with zero-mean and unit variance, *T* is the period of the signal and *A* is the coupling coefficient. The simulation signal in Figure 3a is configurated as *f_1_* = 80 Hz, *f_2_* = 270 Hz, *A* = 1 and *T* = 0.25 *s* with the sampling rate 1,000 Hz, and its wavelet scalogram is shown in Figure 3b.

**Figure 4.**
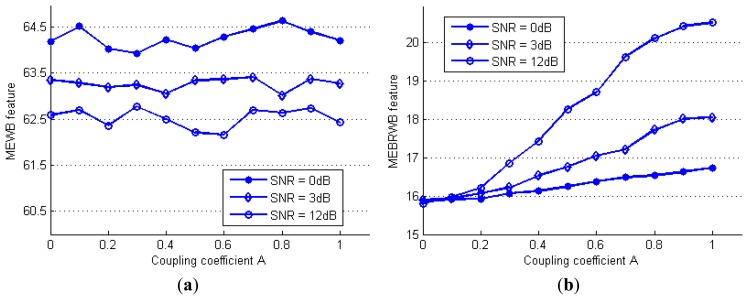
The effect of global QPC on MEWB feature (**a**) and MEBRWB feature (**b**) at different SNR levels.

In this signal, the global QPC energy of the simulation signal is adjusted by increasing the coupling coefficient *A* from 0 to 1. For comparison, the maximum eigenvalue of the traditional WB (MEWB feature) and the proposed MEBRWB feature are estimated using the same data. By changing the coupling coefficient *A* from 0 to 1, the estimation of the MEWB feature and the proposed MEBRWB feature at different SNR levels are obtained and plotted in Figure 4a and Figure 4b, respectively.

It can be seen from Figure 4a that the estimated MEWB feature is increasing as the SNR increases, however, it doesn't change with the increase of the coupling coefficient *A*. Thus, the MEWB feature is not able to detect the coupling components at each SNR levels, when phase-coupled and non-phase-coupled are mixed together. In contrast, as shown in Figure 4b, the estimated MEBRWB feature increases with the increase of the coupling coefficient A, although it decreases with the decreasing SNR levels. In addition, the trend of the MEBRWB feature approximates to linear. Thus the proposed quadratic nonlinearity feature is able to capture changes in global QPC of the signal efficiently, particularly when the SNR is greater than 3 dB.

## 5. Application of Quadratic Nonlinearity Feature for Translational Axis Condition Monitoring of a Vertical Machining Centre

### 5.1. Description of the Experimental System

**Figure 5.**
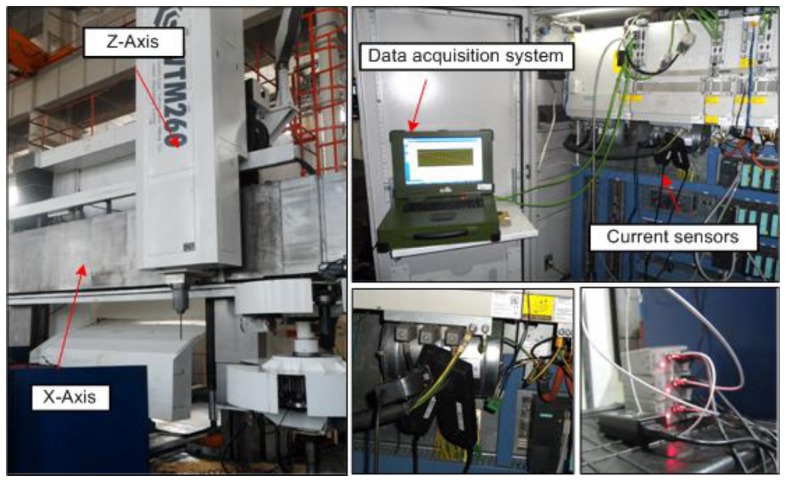
Experimental system.

**Figure 6.**
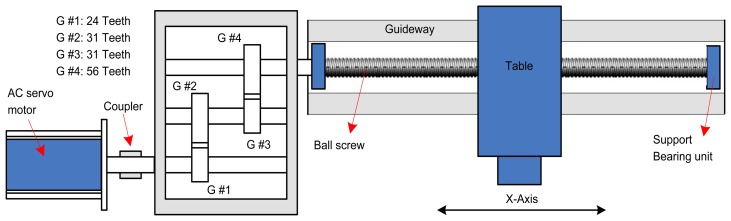
Illustration of the X-axis.

The proposed method is applied to monitor the translational axis of a high precision vertical machining centre. Experiments are conducted on the X- axis of this machining centre in an in-service environment, and the experimental time is the whole maintenance period of the X-axis. The experiment system is shown in Figure 5 and the illustration of the X-axis is shown in Figure 6.

As shown in Figure 6, the translational axis system (X-axis) is composed of an AC servomotor, a reducer, a precision ball screw and a table. The actuation of this system is provided through the AC servomotor which is attached to the reducer using a diaphragm type coupling. The reducer is a three-stage gearbox attached to the precision ball screw also using a diaphragm type coupling (the teeth number of the each gear is shown in Figure 6). The precision ball screw with 16 mm pitch and 40 mm diameter is supported by two bearings, which drives a table supported on a guideway. Experiments are implemented during the whole maintenance period of the X-axis under the condition that the feed rate is 550 mm/min, when the table is fed steadily without cutting. The three-phase AC current signals of the servomotor are measured synchronously by three current sensors, and then, the motor torque is obtained by Equations (1) and (2). The total experimental time is 192 days, and the data is collected with an interval of approximate 26 days. Each data is sampled with sampling frequency 1,000 Hz. Actually, there are eight torque data samples, and each data sample is a collected data series containing 60,000 data points. The characteristic frequencies of the X-axis are given in Table 1.

### 5.2. Experimental Results

**Figure 7.**
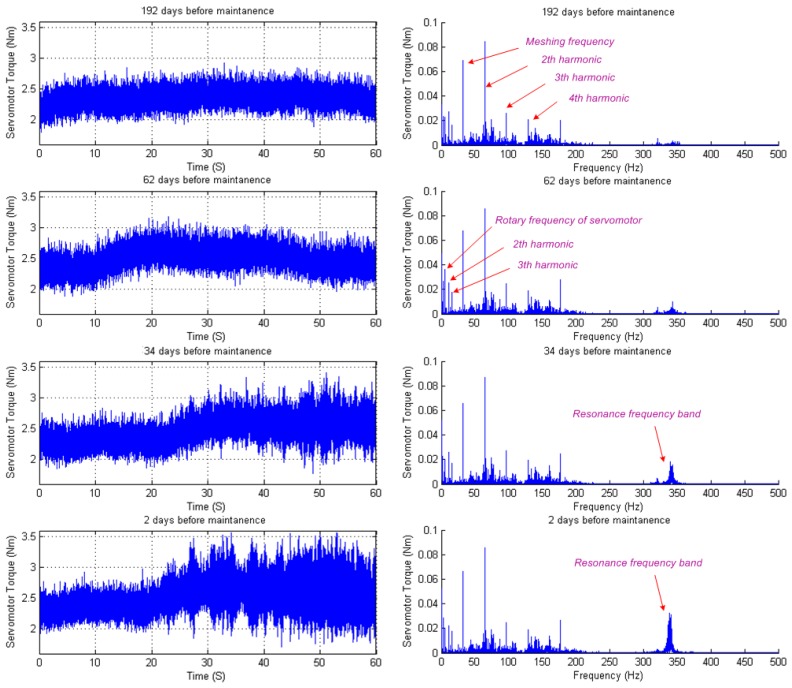
Servomotor torque of X-axis at different number of the days before maintenance.

Figure 7 shows the calculated servomotor torque (left panels) of X-axis and its spectrum (right panels), at 192 days, 62 days 34 days and 2 days before maintenance, respectively. It can be seen from the left panels of Figure 7 that the servomotor torque fluctuates for each data sample. During the last 34 days before maintenance, some impulses appear in the last half of the ball screw travel. As shown in the right panels of Figure 7, the spectrum plot of each data sample shows that the same dominating spectrum peaks appear at the servomotor's rotary frequency and the first, second and third harmonics of the reducer meshing frequency, with very slight changes in the peaks' value. At the same time, a resonance appears at the frequencies band between 330–370 Hz, during the last 62 days before maintenance. Similar results occur in the spectrum of almost every data sample. Thus, it is not easy to describe the degradation progress by directly using the change of the characteristic frequencies.

**Figure 8.**
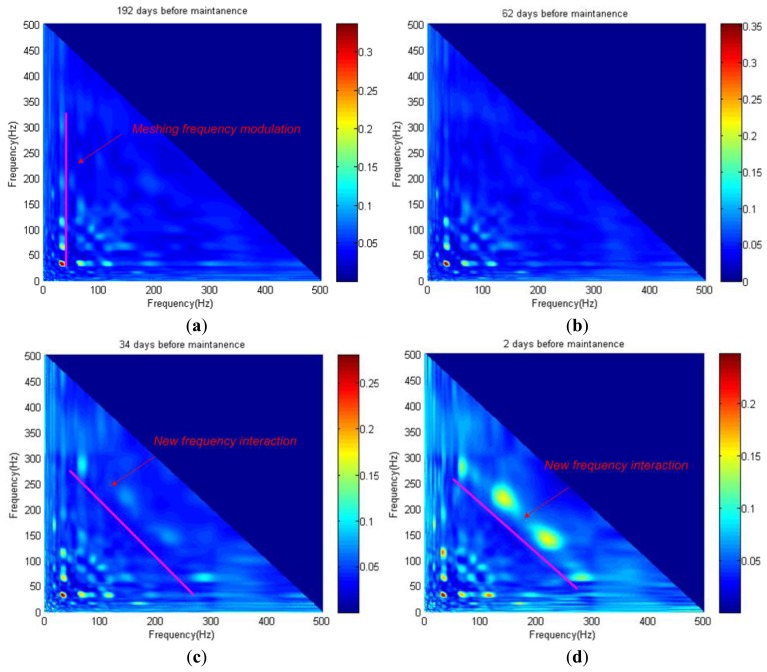
BRWB of X-axis servomotor torque data: (**a**) 192 days before maintenance; (**b**) 62 days before maintenance; (**c**) 34 days before maintenance; (**d**) 2 days before maintenance.

To describe the degradation progress, the proposed BRWB is used to detect the quadratic nonlinear phase coupling frequencies band of the torque signature. Here, the bandwidth from 0–500 Hz is selected for the bicoherence calculation. Figure 8a–d showS the BRWB of the torque data samples at 192 days, 62 days, 34 days and 2 days before maintenance, respectively. As shown in Figure 8a, it is found that the dominant phase coupling peaks appear at the bifrequency (32 Hz, 64–350 Hz), which illustrates that, at the beginning of the service life progress, the quadratic nonlinear interaction only occurs between the 1th, 2th and 3the harmonics of the reducer's meshing frequency. As the service life progresses, new phase coupling presents at the bifrequency (64 Hz, 270 Hz) and (140 Hz, 230 Hz) as shown in Figure 8c,d, which illustrates that quadratic nonlinear interaction between the reducer's meshing frequency and the resonance frequency band results from the degradation of the X-axis. In addition, the peaks value at the new bifrequency (64 Hz, 270 Hz) and (140 Hz, 230 Hz) are increasing as the degradation progresses.

**Figure 9.**
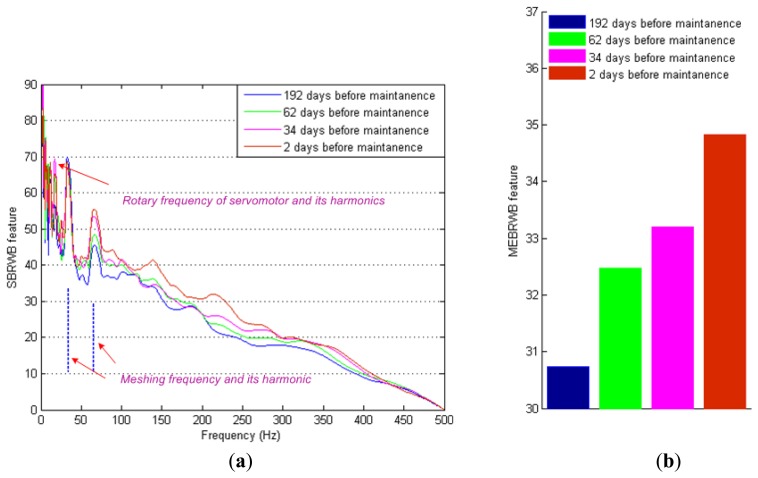
Features of different number of the days before maintenance: (**a**) SBRWB feature; (**b**) MEBRWB feature.

To further investigate the interactions, the phase coupling distribution of each data sample is estimated by using the SBRWB feature, as shown in Figure 9a, and the global phase coupling of each data sample is estimated by the MEBRWB feature as shown in Figure 9b. The differences among the BRWB of each data sample can be directly revealed by the SBRWB feature. The SBRWB features of the resonance frequency band, servomotor rotary frequency harmonics and the second harmonic of reducer meshing frequency are increasing significantly during the service cycle. At the same time, the global phase coupling of each data sample is also increasing significantly during the service cycle, as shown in Figure 9b.

**Figure 10.**
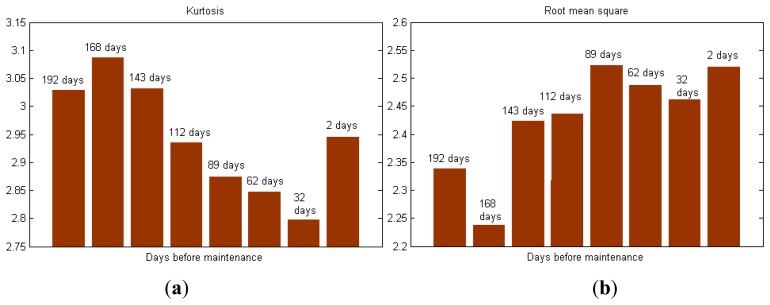

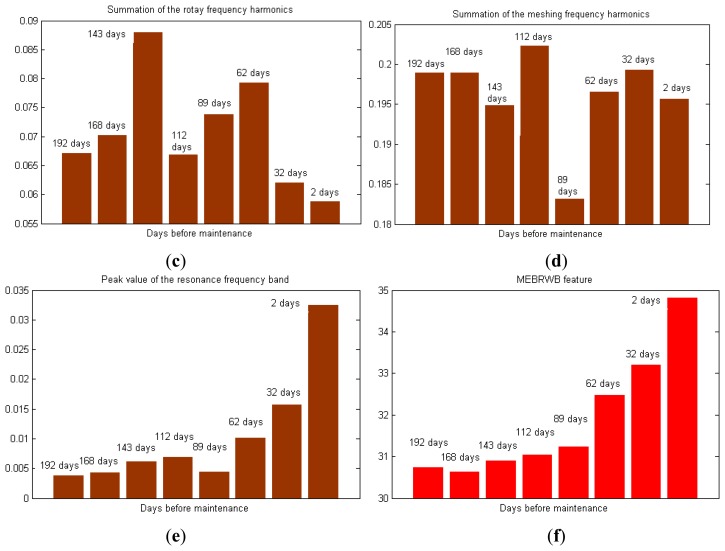
Trend of torque MEBRWB feature compared with the common used features: (**a**) Kurtosis; (**b**) Room mean square; (**c**) Summation of the rotary frequency harmonics; (**d**) Summation of the meshing frequency harmonics; (**e**) Peak value of the resonance frequency band; (**f**) MEBRWB feature.

For convenience, the single-value MEBRWB feature of the eight torque data samples is calculated to monitor the condition of X-axis. The MEBRWB feature is compared with other commonly used feature during the whole maintenance period of the X-axis, as shown in Figure 10. Figure 10a–e shows the kurtosis, root mean square, summation of the rotary frequency harmonics, summation of the meshing frequency harmonics and peak values of the resonance frequency band of the eight measured torque data samples, respectively.

Results show that the commonly used features don't perform well in describing the progress of X-axis degradation. By contrast, as shown in Figure 10f, the value of MEBRWB feature starts to increase at 143 day before maintenance. It keeps increasing until the translational axis system maintenance because of the degradation of the components, which physically can be interpreted as increased additional torque quadratic nonlinear interaction due to the performance degradation of the X-axis. Thus, this trend can be used as precocious indication of performance degradation of the translational axis of the machine tools.

## 6. Conclusions

A BRWB based quadratic nonlinearity feature is established for translational axis condition monitoring of the machine tools, which shows that it is possible to perform condition monitoring by using servomotor torque signature. Some conclusions are drawn as follows:

(1) A BRWB is established to overcome the problem of current WB, which can eliminate the spurious peaks coming from long coherence time waves and non-QPC waves efficiently. Based on the proposed BRWB, a quadratic nonlinearity MEBRWB feature is proposed for translational axis condition monitoring. Numerical example results show that the global QPC of the simulation signal can be tracked approximately linearly by the proposed feature, especially when the SNR is greater than 3 dB.
(2) The proposed quadratic nonlinearity MEBRWB feature is also used to study the torque data collected from a high precision vertical machining centre. Experimental results illustrate the robust experimental performance of the proposed feature, compared with commonly used features. The advantage of MEBRWB feature is that it can exploit the true global QPC of the signal at different frequencies, which contains useful additional information for detection of quadratic nonlinear phenomena induced by mechanical faults. Potentially, this single-valued feature can be used in prognostic models for remanding useful life estimation of mechanical components.

## Figures and Tables

**Table 1.** The characteristic frequencies of X-Axis system.

**Item**	**Characteristic Frequencies**
Feed rate of X-Axis	550 (mm/min)
Rotary frequency of servomotor	5.35 (Hz)
Rotary frequency of idler shaft	1.34 (Hz)
Rotary frequency of output shaft	1.03 (Hz)
Meshing frequency of G#1 and G#2	32.1 (Hz)
Meshing frequency of G#3 and G#4	32.1 (Hz)
Passing frequency of ball screw lead	0.57 (Hz)

## References

[1] Kim M.-S., Chung S.-C. (2006). Friction identification of ball-screw driven servomechanisms through the limit cycle analysis. Mechatronics.

[2] Caesarendra W., Widodo A., Yang B.-S. (2010). Application of relevance vector machine and logistic regression for machine degradation assessment. Mech. Syst. Signal Process..

[3] Zhou Y., Mei X., Jiang G., Sun N., Tao T. (2010). Sensorless evaluation for a computer numerical control machine tool slide level using an empirical mode decomposition method. Proc. Inst. Mech. Eng. Part C: J. Mech. Eng. Sci..

[4] Chen G., Yuan J., Ni J. (2001). A displacement measurement approach for machine geometric error assessment. Int. J. Mach. Tools Manuf..

[5] Whalley R., Ebrahimi M., Abdul-Ameer A.A. (2005). Hybrid modelling of machine tool axis drives. Int. J. Mach. Tools Manuf..

[6] Yu J.-B. (2011). Bearing performance degradation assessment using locality preserving projections. Expert Syst. Appl..

[7] Hassan M.A., Bayoumi A.M.E., Shin Y.J. (2013). Quadratic-nonlinearity index based on bicoherence and its application in condition monitoring of drive-train components. IEEE Trans. Instrum. Meas..

[8] Huang R., Xi L., Li X., Richard Liu C., Qiu H., Lee J. (2007). Residual life predictions for ball bearings based on self-organizing map and back propagation neural network methods. Mech. Syst. Signal Process..

[9] Qiu H., Lee J., Lin J., Yu G. (2006). Wavelet filter-based weak signature detection method and its application on rolling element bearing prognostics. J. Sound Vib..

[10] Qiu H., Lee J., Lin J., Yu G. (2003). Robust performance degradation assessment methods for enhanced rolling element bearing prognostics. Adv. Eng. Inform..

[11] Ottewill J.R., Orkisz M. (2013). Condition monitoring of gearboxes using synchronously averaged electric motor signals. Mech. Syst. Signal Process..

[12] Hwang K.H., Lee J.M., Hwang Y. (2013). A new machine condition monitoring method based on likelihood change of a stochastic model. Mech. Syst. Signal Process..

[13] Sinha J.K., Elbhbah K. (2013). A future possibility of vibration based condition monitoring of rotating machines. Mech. Syst. Signal Process..

[14] Yu J. (2013). A nonlinear probabilistic method and contribution analysis for machine condition monitoring. Mech. Syst. Signal Process..

[15] Lei Y., Kong D., Lin J., Zuo M.J. (2012). Fault detection of planetary gearboxes using new diagnostic parameters. Meas. Sci. Technol..

[16] Nichols J.M., Olson C.C. (2010). Optimal bispectral detection of weak, quadratic nonlinearities in structural systems. J. Sound Vib..

[17] Nichols J.M., Marzocca P., Milanese A. (2009). The trispectrum for Gaussian driven, multiple degree-of-freedom, non-linear structures. Int. J. Non-Linear Mech..

[18] Gu F., Shao Y., Hu N., Naid A., Ball A.D. (2011). Electrical motor current signal analysis using a modified bispectrum for fault diagnosis of downstream mechanical equipment. Mech. Syst. Signal Process..

[19] Kim G.D., Chu C.N. (1999). Indirect cutting force measurement considering frictional behaviour in a machining centre using feed motor current. Int. J. Adv. Manuf. Technol..

[20] Jeong Y.-H., Cho D.-W. (2002). Estimating cutting force from rotating and stationary feed motor currents on a milling machine. Int. J. Mach. Tools Manuf..

[21] Mei X., Tsutsumi M., Tao T., Sun N. (2003). Study on the load distribution of ball screws with errors. Mech. Mach. Theory.

[22] Verl A., Frey S. (2010). Correlation between feed velocity and preloading in ball screw drives. CIRP Ann. Manuf. Technol..

[23] Fackrell J., McLaughlin S., White P. (1995). Practical Issues Concerning the Use of the Bicoherence for the Detection of Quadratic Phase Coupling.

[24] Courtney C.R.P., Neild S.A., Wilcox P.D., Drinkwater B.W. (2010). Application of the bispectrum for detection of small nonlinearities excited sinusoidally. J. Sound Vib..

[25] Peng Z.K., Zhang W.M., Yang B.T., Meng G., Chu F.L. (2013). The parametric characteristic of bispectrum for nonlinear systems subjected to Gaussian input. Mech. Syst. Signal Process..

[26] Gelman L., White P., Hammond J. (2005). Fatigue crack diagnostics: A comparison of the use of the complex bicoherence and its magnitude. Mech. Syst. Signal Process..

[27] Elsayed M.A.K. (2006). Wavelet bicoherence analysis of wind–wave interaction. Ocean Eng..

[28] Taplidou S.A., Hadjileontiadis L.J. (2007). Nonlinear analysis of wheezes using wavelet bicoherence. Comput. Biol. Med..

[29] Li X., Li D., Voss L.J., Sleigh J.W. (2009). The comodulation measure of neuronal oscillations with general harmonic wavelet bicoherence and application to sleep analysis. NeuroImage.

[30] Van Milligen B.P., Sanchez E., Estrada T., Hidalgo C., Branas B., Carreras B., Garcia L. (1995). Wavelet bicoherence: A new turbulence analysis tool. Phys. Plasmas.

[31] Combet F., Gelman L., LaPayne G. (2012). Novel detection of local tooth damage in gears by the wavelet bicoherence. Mech. Syst. Signal Process..

[32] Li Y., Lin J., Wang X., Lei Y. (2014). Biphase randomization wavelet bicoherence for mechanical fault diagnosis. Measurement.

[33] Taekhyun K., Powers E.J., Grady W.M., Arapostathis A. A Novel QPC Detector for the Health Monitoring of Rotating Machines.

[34] Li X., Yao X., Fox J., Jefferys J.G. (2007). Interaction dynamics of neuronal oscillations analysed using wavelet transforms. J. Neurosci. Methods.

[35] Lin J. (2001). Feature extraction of machine sound using wavelet and its application in fault diagnosis. NDT E Int..

